# An Electrostatic MEMS Roll-Pitch Rotation Rate Sensor with In-Plane Drive Mode

**DOI:** 10.3390/s22030702

**Published:** 2022-01-18

**Authors:** Ahmed Khaled, Ahmed M. Salman, Nawaf S. Aljehani, Ibrahim F. Alzahem, Ridha S. Almikhlafi, Radwan M. Noor, Yasser M. Seddiq, Majed S. Alghamdi, Mostafa Soliman, Mohamed A. E. Mahmoud

**Affiliations:** 1 Si-Ware Systems, Heliopolis, Cairo 11361, Egypt; ahmed.khaled@si-ware.com (A.K.); ahmed.mohamed.mostafa@eng.asu.edu.eg (A.M.S.); mostafa.soliman@si-ware.com (M.S.); 2 Mechatronics Engineering Department, Ain Shams University, Cairo 11535, Egypt; 3 Communication and Information Technology Research Institute, King Abdulaziz City for Science and Technology (KACST), Riyadh 12354, Saudi Arabia; naljehani@kacst.edu.sa (N.S.A.); ialzahem@kacst.edu.sa (I.F.A.); ralmikhlafi@kacst.edu.sa (R.S.A.); yseddiq@kacst.edu.sa (Y.M.S.); 4 Electronics and Electrical Communication Engineering, Ain Shams University, Cairo 11535, Egypt

**Keywords:** MEMS, gyroscope, roll-pitch, ASIC, FEM, capacitive, monolithic, design methodology

## Abstract

In this paper, we presented a novel electrostatic Roll/Pitch MEMS gyroscope with in-plane drive mode and out-of-plane sense mode. The proposed structure is developed based on a tuning fork gyroscope with decoupled sense mass on each tine that control the sense out-of-plane frequency. A multi-height deep reactive ion etching (DRIE) fabrication process was utilized to achieve and enhance decoupling between the drive and sense modes. We presented our design methodology followed by an analytical and finite element (FEM) model. Our experimental results showed a good match between the analytical model and those obtained experimentally, from the drive and sense oscillation frequencies. Our characterization setup used a custom made application specific integrated circuit (ASIC) for characterization and was able to achieve ARW of 0.2 deg/rt-h, a bias instability 5.5 deg/h, and scale factor non-linearity (SFNL) 156 ppm FS.

## 1. Introduction

Multiple axes Micro Electro-Mechanical Systems (MEMS) gyroscopes have dominated the consumer electronics market due to their small size, low cost and fast startup time. Most of these sensors can measure rotation rate in multiple directions using a single mechanical element which yields miniaturized and compact sensors. Nevertheless, this approach compromises the multiple axes gyro performance and limits it to consumer applications [1,2,3,4].

To overcome these limitations and to allow the MEMS gyro to be utilized in tactical and industrial applications, researchers adopted different approaches. These approaches ranges from discrete assembly of high performance individual gyro sensors [5,6,7,8], to folded 3D Inertial Measurement Unit (IMU) sensors [9], all the way to monolithic fabrication of multiple in-plane and out-of-plane detection sensors with separate mechanical elements [10]. The common factor of all of these approaches is the use of single mechanical element per axis which reduces the cross-axis sensitivity and ensures the expected high performance.

Discrete assembly is done by placing individual MEMS gyro sensors on Printed Board Circuits (PCBs) and aligning them in an orthogonal manner. These sensors are usually high performance single axis gyro sensors that measure dominantly the effect of rotation in the *z*-axis (out-of-plane) with tine in-plane motion detection. This is the state of the art approach and it delivers the highest performance MEMS inertial gyro sensors [5,6,7,8]. The folded 3D IMU approach combines fabricating high performance single axis sensors in-situ with the flexible hinges lithographically defined latches [9]. This approach requires complicated assembly steps to ensure the sensors are in a 3D IMU configuration, which adds up to the manufacturing complexity and introduces additional errors that may or may not be possible to calibrate out. Monolithic fabrication of in-plane and out-of-plane sensors can yield compact sensors and is immune to post fabrication assembly errors. The limiting factor of this approach is the in-plane sensitive sensors (Roll/Pitch) due to the need to either out of plane drive or detection. One approach is to drive out-of-plane and detect both Roll and/or Pitch in-plane [11]. However, this approach suffers from the elevated squeeze film damping leading to reduce the sensor out-of plane motions and thereby its quality factor. It also has an effect of reducing the Coriolis force and Signal to Noise Ratio (SNR) which leads to deteriorated sensor performance. Other approach which is adopted in this work is to drive in-plane instead of out-of-plane in order to minimize the effect of squeeze film damping, thereby achieving long drive strokes, high capacitance change rate, and high SNR of the gyro output.

In this paper, we present a Roll/Pitch MEMS gyroscope design with in-plane drive mode and out-of-plane sense mode. The proposed structure is developed as a tuning fork gyroscope with decoupled sense mass on each tine that can control the sense out-of-plane frequency. This is enabled by multi-height DRIE process. A similar design was reported in [12], however on a single tine structure.

This paper is structured as follows. In Section 2.1, we briefly described the followed design methodology of this roll/pitch MEMS gyro sensor. Then, in Section 2.3, we introduced both the analytical and the finite element models and, subsequently, supported results of such analyses by experimental validations. In Section 2.4, we go over the gyroscope fabrication process. Section 3.1 represents the experimental setup used to characterize the sensor. Section 3.2 highlights the experimental results and presents a discussion of those results. Section 4 concludes our work and briefly goes over future work.

## 2. Materials and Methods

### 2.1. Sensor Modeling

#### 2.1.1. Unmatched Tuning Fork Gyroscopes

**Figure 1:**
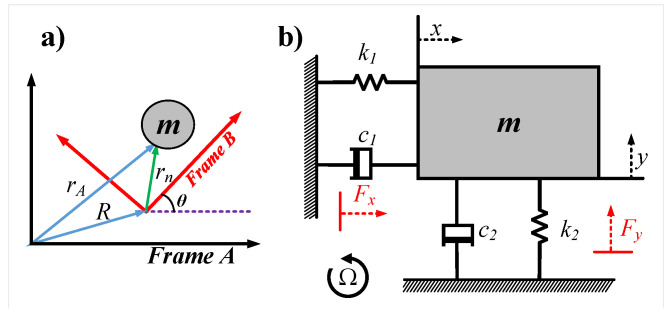
(**a**) Gyroscope frame B rotating in a reference with respect to inertial frame A. (**b**) Schematic diagram of the gyroscope lumped mass model.

The fundamental operation of Vibratory Coriolis Gyroscope devices depend on the Coriolis force principal [13,14]. As shown in Figure 1a, a vibrating mass in the rotating gyroscope frame (frame B) relative to the inertial frame of reference (frame A) will experience a fictitious force called Coriolis force [13,14,15] given by:

(1) $$F_{c}=2m\Omega \times v_{b},$$

where $F_{c}$ is the Coriolis force, *m* is the lumped gyroscope mass, Ω is the gyroscope angular velocity, and $v_{b}$ is the local velocity of the mass in the gyroscope frame. This force can be sensed to measure the angular velocity of the gyroscope. To formulate the equations of motions, we consider lumped mass model of the gyroscope as shown in Figure 1b. The Coriolis force experienced acts in the *y*-axis (sense direction) since the mass is driven in *x*-axis (drive direction) and the rotation is about the *z*-axis (rotation direction). A simplified equations of motion can be formulated as follows [16]:

(2) $$\ddot{x}+c_{x}\dot{x}+k_{x}x=F_{d},$$

(3) $$\ddot{y}+c_{y}\dot{y}+k_{y}y=F_{c},$$

where $F_{d}$ is the drive force, $c_{x}$ and $c_{y}$ are the damping factors in *x* and *y* directions, respectively, and $k_{x}$ and $k_{y}$ are the stiffnesses in *x* and *y* directions, respectively. In these equations, we assume nearly constant angular rate (${\dot{\Omega }}_{x}\approx 0$) and angular rates at much lower frequencies than the operating frequency of the gyroscope ($\Omega_{x}^{2},\Omega_{y}^{2}\approx 0$) [14]. Equations (2) and (3) are second order differential equations, suggesting the presence of both a drive and sense modes. The gyroscope is operated in the drive direction at the drive frequency and (1) dictates that the Coriolis force has the same frequency. To get the maximum displacement in the sense direction, it is required to have a matching drive and sense frequencies, however, unmatched frequency operation was preferred because it eliminates the need for high end electronics required to lock both drive and sense frequencies to be the same.

Gyroscopes can be designed as a lumped mass, single mass system, or a coupled two-mass system, known as tuning fork gyroscopes (TFG). In the two-mass systems, the masses are driven in opposite directions to provide a differential sensing signal which minimize common mode excitation like environmental vibrations and increases the gyroscope sensitivity, so it was chosen for our design. However, this gives rise to the undesirable in-phase modes in addition to the out-of-phase mode in both drive and sense directions, which are studied in the following subsections.

#### 2.1.2. Drive Stiffness Network Analysis

**Figure 2:**
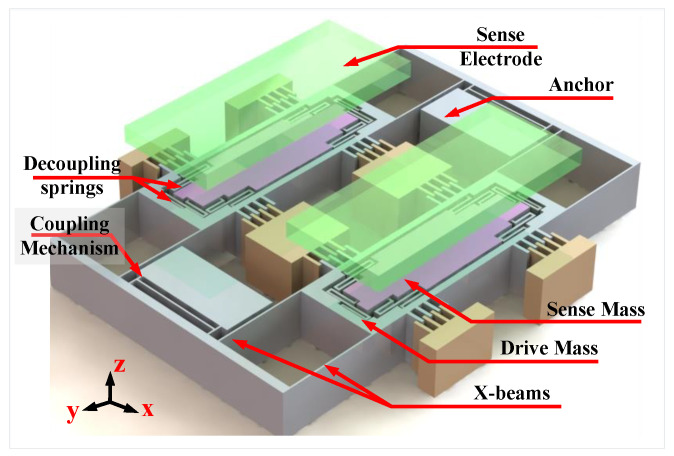
3D model of the proposed gyroscope design.

**Figure 3:**
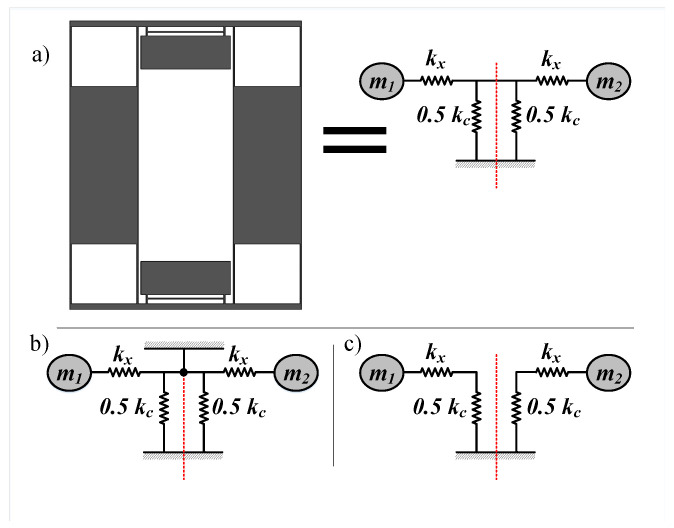
Drive stiffness network analysis. (**a**) Simplified TFG design and its equivalent mass-stiffness network in drive direction, with the coupling stiffness split into two parallel stiffnesses (**b**) The out-of-phase mode where the red line is a virtual anchor (**c**) The in-phase mode where any connection on the red line can be opened because both masses are in an in-phase motion.

The proposed structure for our TFG is shown in Figure 2. Each side consists of four fixed-fixed beams (X-beams) from each side to act as springs in the drive directions (X-spring), an outer frame, an inner mass, and six serpentine structures (Z-serpentine) to act springs in the sense direction (Z-spring). In this structure, the two masses are driven in opposite direction and are coupled by two symmetric upper and lower coupling mechanisms in order to ensure the coupled operation. The rotation axis is x directions and the Coriolis force acts in the z direction deferentially on both masses which act as capacitor plates used to sense this force. To understand the nature of the coupling and calculate the in-phase and out-of-phase drive frequencies, the network is analyzed as shown in Figure 3, splitting the coupling stiffness to two parallel stiffnesses to be able to perform in-phase and out-of-phase analyses. Since the masses are moved together in the same direction, the in-phase mode, the link between them in the network is insignificant and can be removed as shown in Figure 3c. In consequence, the in-phase mode drive frequency can be formulated as:

(4) $$f_{d_{in}}=\sqrt{\frac{K_{d_{in}}}{M}},$$

where $K_{d_{in}}$ can be formulated as:

(5) $$K_{d_{in}}=\frac{K_{x}K_{c_{x}}}{2K_{x}+K_{c_{x}}},$$

where $f_{d_{in}}$ is the in-phase drive frequency, $K_{d_{in}}$ is the in-phase drive stiffness, *M* is the effective mass of each tile, $K_{x}$ is the stiffness of the X-beams and $K_{c_{x}}$ is the stiffness of the coupling mechanism in the *x*-axis direction.

However, in the case of the out-of-phase motion, that link is essentially not moving and can be treated as a virtual anchor as shown in Figure 3b. Consequently, the out-of-phase frequency can be formulated as:

(6) $$f_{d_{out}}=\sqrt{\frac{K_{d_{out}}}{M}},$$

where $K_{d_{out}}$ can be formulated as:

(7) $$K_{d_{out}}=K_{x},$$

where $f_{d_{out}}$ is the out-of-phase drive frequency and $K_{d_{out}}$ is the out-of-phase drive stiffness.

It is evident that the out-of-phase mode frequency is higher, and the difference between the in-phase and out-of-phase drive modes increases as the coupling stiffness decreases. This serves as the main parameter to control the drive modes frequency difference.

#### 2.1.3. Sense Stiffness Analysis

**Figure 4:**
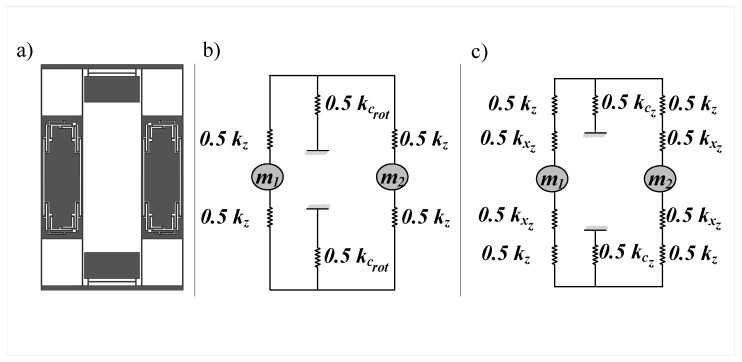
(**a**) Top view of the gyroscope structure (**b**) Equivalent mass-stiffness network in the sense direction for the out-of-phase mode (**c**) Equivalent mass-stiffness network in the sense direction for the in-phase mode.

The out-of-phase sense mode stiffness is primarily determined by the torsional stiffness of the coupling mechanism and the internal Z-springs shown in Figure 4a, and its frequency can be formulated as:

(8) $$f_{s_{out}}=\sqrt{\frac{K_{s_{out}}}{M}},$$

where $K_{s_{out}}$ can be formulated as:

(9) $$K_{s_{out}}=\frac{K_{c_{rot}}K_{z}}{K_{c_{rot}}+K_{z}},$$

where $f_{s_{out}}$ is the out-of-phase sense frequency, $K_{z}$ is Z-springs stiffness, $K_{s_{out}}$ is the out-of-phase sense stiffness and $K_{c_{rot}}$ is the torsional stiffness of the coupling mechanism.

However, the in-phase sense mode stiffness is the series combination of the Z-springs stiffness, vertical stiffness of the X-beams and vertical stiffness of the coupling mechanism as shown in Figure 4b and its frequency can be formulated as:

(10) $$f_{s_{in}}=\sqrt{\frac{K_{s_{in}}}{M}},$$

where $K_{s_{in}}$ can be formulated as:

(11) $$K_{s_{in}}=\frac{K_{z}K_{x_{z}}K_{c_{z}}}{K_{z}K_{x_{z}}+K_{x_{z}}K_{c_{z}}+K_{z}K_{c_{z}}},$$

where $f_{s_{in}}$ is the in-phase sense frequency, $K_{s_{in}}$ is the in-phase sense stiffness, $K_{x_{z}}$ is the vertical stiffness of the x beams, $K_{c_{z}}$ is the vertical stiffness of the coupling mechanism. Since the vertical stiffnesses of the beams in the vertical direction are typically much higher than the torsional stiffness, the in-phase mode is expected to have a higher frequency than the in-phase mode. Owing to this and based on our conclusions from Section 2.1.2 that the out-of-phase drive mode is higher than the in-phase one, it is possible to achieve the correct mode ordering where the out-of-phase sense mode is directly after the out-of-phase drive mode.

#### 2.1.4. Design Methodology

**Figure 5:**
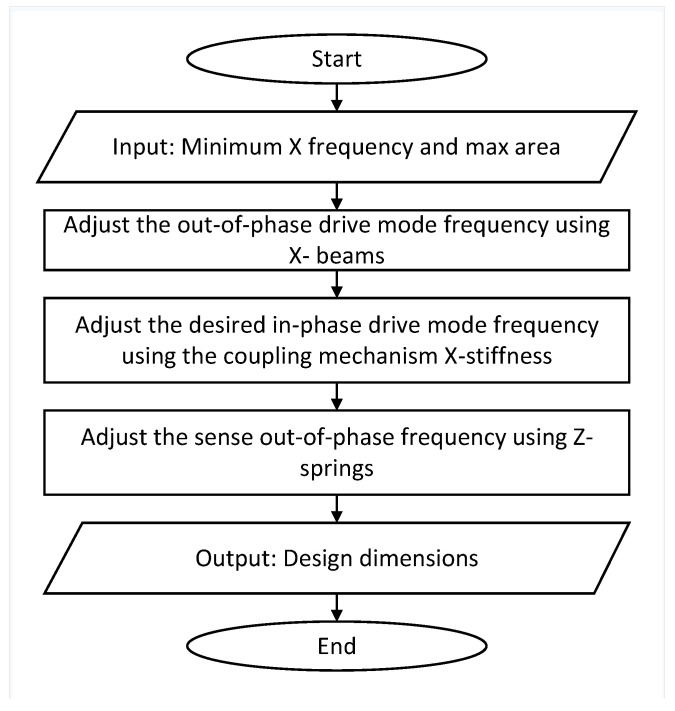
Flow chart of TFG design methodology.

To achieve the correct mode ordering and separations of the in- and out-of-phase drive and sense modes we followed the design methodology presented by Figure 5.

First, we begin with the maximum allowed gyroscope area and minimum operating frequency range. It is advised to design the mass to be as large as possible to ensure higher coriolis force and larger sense capacitance electrode in order to maximise the sensitivity. However, the frequency should be higher than the ambient vibrations frequency range to minimize the effect of vibration on the gyroscope output. As (7) shows, we begin adjusting the X-beams stiffness to get the required out-of-phase drive mode frequency. After that, we adjust the coupling mechanisms x-stiffness to have the desired in-phase drive mode frequency where increasing this stiffness decreases the difference between the in- and out-of-phase frequencies. Next, we adjust the sense out-of-phase frequency using the Z-springs and the inner mass dimensions. The in-phase sense mode is naturally higher because it depends on vertical stiffnesses as shown in (11), which are higher than the torsional stiffness of the coupling mechanism for typical beam dimensions.

### 2.2. Angular Rate Sensitivity Estimation

**Figure 6:**
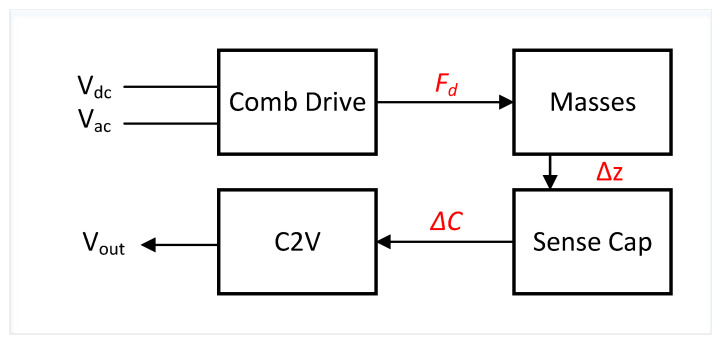
High level electro-mechanical model to calculate the TFG sensitivity using basic electrical and mechanical parameters.

The gyroscope can be electro-mechanically modelled as shown in Figure 6. First, the gyroscope masses are driven using comb drive actuators. The motion of the masses are out-of-phase for proper drive operation mode. The electrostatic force for the comb finger is given by:

(12) $$F_{d}=\frac{1}{2}\frac{\partial C_{x}}{\partial x}V^{2},$$

where $F_{d}$ is the drive force exerted by the comb in one side of the mass, $C_{x}$ is the comb capacitance of one side of the mass, *x* is the displacement and *V* is the applied voltage. In addition to the out-of-phase operation, we have push-pull comb fingers per mass. This configuration is actuated by voltages that are out-of-phase to increase the amount of forcing.

**Figure 7:**
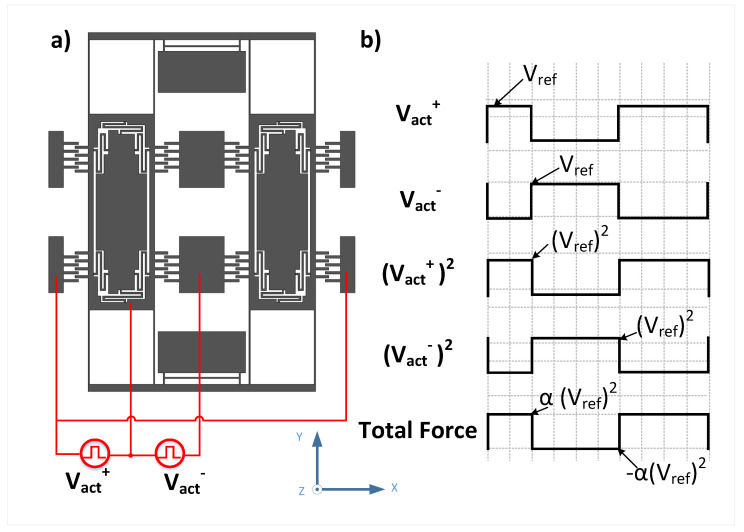
(**a**) The voltage application on both mass ends (**b**) The excitation signal wave-forms of the applied voltage.

One way of applying the voltage is shown in Figure 7. The voltage is applied as a square wave with variable duty cycle and an amplitude of $V_{ref}$ as maximum value while 0V as minimum value. So the resultant force on the mass is also a square wave but with an amplitude proportional to $V_{ref}^{2}$ as maximum value while zero N as minimum value and with the same duty cycle. Moreover, we have the voltage signal to be out-of-phase at the two sides of the mass.

The duty cycle of the voltage and thus the force is generated through a ΣΔ modulator bit stream. This force bit stream can be modeled as a sinusoidal signal at frequency $f_{d}$ with amplitude value α of $V_{ref}^{2}$ in addition to a shaped noise that will be filtered by the moving MEMS that act as band-pass filter for the ΣΔ bit stream. Moreover, the value of α will be determined by the density of bit stream which will be controlled by an Automatic Gain Control (AGC) block within the ΣΔ modulator. The force on each mass can be expressed then as follows:

(13) $$F_{d}=\frac{1}{2}\frac{\partial C_{x}}{\partial x}\alpha V_{ref}^{2}\left(\cos\left(2\pi f_{d}t\right)+n_{hf}\right),$$

where α is the ΣΔ scaling factor, $f_{d}$ is the frequency of the drive voltage that is the same as the MEMS resonance frequency, $n_{hf}$ is the high frequency noise force component due to ΣΔ modulator. As we can clearly see, the applied force is controlled by changing the scaling factor α while applying a constant reference voltage. Since the MEMS will filter out the input frequencies except the one at the resonance frequency, the high frequency noise can be neglected and the applied force can be simplified to:

(14) $$F_{d}=\frac{1}{2}\frac{\partial C_{x}}{\partial x}\alpha V_{ref}^{2}\cdot \cos\left(2\pi f_{d}t\right),$$

The comb capacitance can be formulated as:

(15) $$C_{x}=2N\frac{\epsilon_{0}\left(x_{ov}+x\right)d}{g_{x}},$$

where *N* is the number of drive actuation comb fingers in each side of the mass, $\epsilon_{0}$ is the free space permettivity, $x_{ov}$ is the finger overlap, *x* is the displacement in the drive direction, *d* is the comb thickness, and $g_{x}$ is the finger gap.

The differentiation of the capacitor with respect to the displacement can be formulated as:

(16) $$\frac{\partial C_{x}}{\partial x}=2N\frac{\epsilon_{0}d}{g_{x}}.$$

The displacement obtained at resonance is approximately calculated using the x-stiffness:

(17) $$x=Q\frac{F_{d}|max}{K_{d_{out}}}sin\left(2\pi f_{d}t\right)=x_{0}sin\left(2\pi f_{d}t\right),$$

with $x_{0}$ is:

(18) $$x_{0}=NQ\frac{\epsilon_{0}d}{g_{x}K_{d_{out}}}\alpha V_{ref}^{2}.$$

where *Q* is the drive-mode quality factor and $F_{d}|max$ is the amplitude of driving force. Note that due to the 90 degree phase shift between the force and displacement at resonance, the cosine in the force expression has changed to sine in the displacement expression. The Coriolis force experienced is given by:

(19) $$F_{c}=-2m\Omega \frac{dx}{dt},$$

where *x* is given by (17). This yields:

(20) $$F_{c}=-4\pi m\Omega x_{0}f_{d}cos\left(2\pi f_{d}t\right),$$

and this force acts on the vertical capacitor plates, yielding the following vertical displacement as a displacement due to the Coriolis force $F_{c}$:

(21) $$z=\left|H\frac{F_{c}}{K_{s_{out}}}\right|=H\frac{4\pi m\Omega x_{0}f_{d}cos\left(2\pi f_{d}t\right)}{K_{s_{out}}},$$

where *H* is the sense direction transfer function. Since the drive and sense resonance frequencies are unmatched, *H* can be given by:

(22) $$H=\frac{1}{\sqrt{{\left(1-{\left(\frac{f_{d}}{f_{s}}\right)}^{2}\right)}^{2}+{\left(\frac{f_{d}}{f_{s}Q_{s}}\right)}^{2}}},$$

where $f_{s}$ is the sense frequency and $Q_{s}$ is the sense quality factor. This vertical displacement can be converted to a capacitance signal:

(23) $$\Delta C=C_{+}-C_{-}=\epsilon_{0}A\left(\frac{1}{g_{z}-z}-\frac{1}{g_{z}+z}\right)=\frac{2\epsilon_{0}Az}{g_{z}^{2}-z^{2}},$$

where $C_{+}$ and $C_{-}$ are the capacitance of the rising and falling masses, respectively, *A* is the capacitor area and, $g_{z}$ is the nominal gap between the moving mass and top electrode. Since the vertical displacement is very small compared to the gap, the capacitance signal can be very well approximated as:

(24) $$\Delta C≃\frac{2\epsilon_{0}Az}{g_{z}^{2}}≃H\frac{2\epsilon_{0}A}{g_{z}^{2}}\frac{4\pi m\Omega x_{0}f_{d}cos\left(2\pi f_{d}t\right)}{K_{s_{out}}},$$

We can use the peak amplitude of the capacitance signal ΔC as an estimate for the sensitivity of the gyroscope toward angular velocity and make it as one of the design target parameters and hence the angular rate sensitivity can be deduced:

(25) $$S=\frac{\Delta C}{\Omega }=H\frac{8\pi \epsilon_{0}mAx_{0}f_{d}}{g_{z}^{2}K_{s_{out}}},$$

where *S* is the sensitivity with the units of capacitance per degree per second. The sensitivity can be also expressed in terms of drive-mode natural frequency as follows:

(26) $$S=H\frac{2\epsilon_{0}Ax_{0}f_{d}}{\pi g_{z}^{2}f_{s}^{2}}.$$

This expression will be evaluated and compared to FEM simulation results in Section 2.3.3 and compared to the measurement results in Section 3.2.

### 2.3. Design and Simulation

#### 2.3.1. Physical Design and FEM Model

Based on the methodology described in Figure 5, we need first to choose both the minimum operating frequency range and the allowed gyro area as design inputs. Operating frequencies higher than 7 kHz should be targeted for many applications as it will be high enough for the ambient vibrations frequency range [17]. In addition, the area available for our gyroscope was set to about 5 mm × 5 mm.

We are using fixed-fixed beams in the drive direction which are known to be subject to some non-linearity for long travelled displacements [18]. Based on the beams length, we will target a drive displacement of only 10 μm. As for the sensing capacitor gap, it is chosen to be 4 μm. This choice is based on design judgment to compromise between increasing the sense electrode sensitivity and the risk of stiction during fabrication.

Now, the only parameter we need to figure out is the target frequency difference between sense and drive modes since our gyro works in an unmatched operation mode. To determine the suitable frequency difference, we have to specify the target sensitivity. The ASIC used for the interface electronics recommend a sensitivity of about 40 aF per degree per second (dps) to able to support a full scale angular velocity of ±300 dps while leaving a slack in the ASIC dynamic range for the quadrature.

Substituting all of these values in (26), we deduced that a good target frequency difference will be around 600 Hz. Starting from these inputs, the design was tuned according to the aforementioned methodology and the final design results are presented in the following subsections.

**Figure 8:**
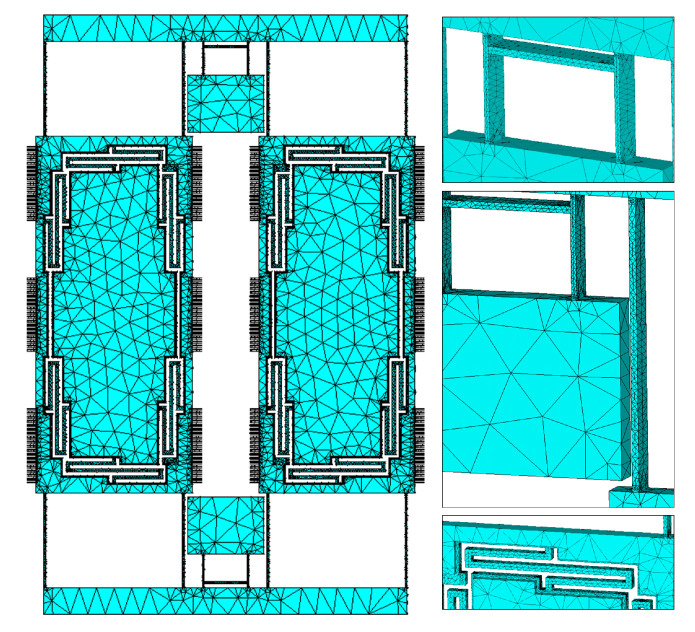
FE mesh elements and nodes. The inserts on the right show finer meshing on the suspensions compared to the proof mass.

This design was modeled using FEM method on ANSYS Mechanical APDL software, the used meshing element is a 3D Tetrahedral solid element with 10 nodes (SOLID187), with the total number of 291K elements in the design. The mesh size was determined using the smart sizing feature so that the mesh size is adjusted automatically at each location of the design. In addition, the meshing was refined at all of the beams location as these are narrow and greatly affect the frequency results. To ensure that the mesh is small enough, mesh accuracy exercise was performed where we changed the mesh accuracy level and observed the first four modes frequencies until the results converged. The meshing at multiple location using the final mesh setting is shown in Figure 8.

#### 2.3.2. Modal Analysis and Spring Linearity

**Figure 9:**
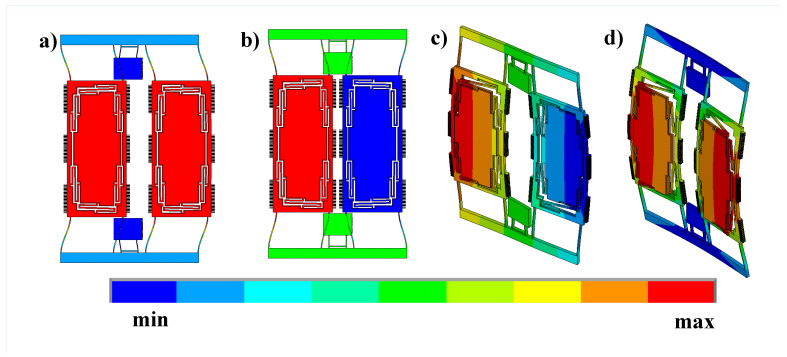
The first four mode shapes of the design (**a**) First drive in-plane (in-phase) mode shape. (**b**) Second drive in-plane (out-of-phase). (**c**) First sense out-of-plane (out-of-phase) mode shape. (**d**) Second sense out-of-plane (in-phase) mode shape.

Modal analysis was performed on our model as discussed in the previous subsection, and mode shapes are reported in Figure 9 with the corresponding frequencies shown in Table 1. As we can see, the modes are arranged correctly, with about 600 Hz frequency difference between the out-of-phase sense and drive modes. Due to fabrication imperfections, we need to perform tolerance analysis across our corners because if the sense mode precedes the drive mode, the gyroscope can not work as intended. The DRIE mask tolerance in the process used is about ±100 nm per edge, so tolerance analysis performed using this number is reported in Table 2. As shown, the worst corner was with +100 nm per edge, with a frequency difference of almost 500 Hz, showing that the design corners are good enough; as they are still correctly ordered with reasonable frequency separation between the out-of-phase drive and out-of-phase sense modes.

Drive spring linearity was calculated using static non-linear analysis for a low displacement of 1 μm and at the full scale displacement of 10 μm yielding 408 N/m and 439 N/m drive spring constants, respectively; hence, the drive spring non linearity is estimated to be about 7.6%. As for the sense spring, the springs are linear because of the limited displacement in the sense direction as will be shown in the following subsections. The sense spring stiffness was found to be 445 N/m.

#### 2.3.3. Scale Factor Calculation

**Figure 10:**
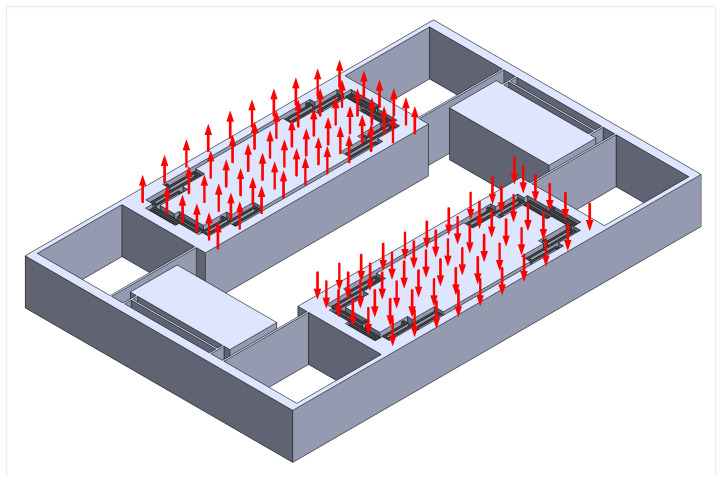
The application style of coriolis force on the gyroscope. The force is distributed across nodes.

In order to calculate the scale factor using FEM, Coriolis force for one dps input was worked out by hand then fed to the simulator. The Coriolis force for the inner mass, outer frame and Z-springs were calculated using (19) and applied separately in a differential manner as shown in Figure 10. It was found out that the displacement of the right end of each inner mass is different than the left displacement of the same inner mass, which is expected since the coupling mechanism has a torsional motion in the sense out-of-phase mode, so the mass tiles motion has a rotational part. The displacement of each inner mass ends were found to be 52.5 and 32.5 pm, so the capacitor formed by each inner mass is a tilted plate capacitor, not a parallel plate one, so we need to use capacitance change formula for a tilted plate capacitor given by:

(27) $$C_{\pm_{tilt}}=\epsilon_{0}A\frac{\pm 1}{z_{1}-z_{2}}ln\left(\frac{g_{z}\mp z_{2}}{g_{z}\mp z_{1}}\right),$$

where $C_{\pm_{tilt}}$ is the capacitance for a tilting plate capacitor for each of the rising and falling plates, $z_{1}$ and $z_{2}$ are the displacements of both mass ends. However, that capacitance can be very well approximated for small displacements using the parallel plate capacitance change formula, which was used to formulate the sensitivity model discussed in Section 2.2, while using the average displacement of both mass ends:

(28) $$C_{\pm_{parallel}}=\epsilon_{0}A\left(\frac{1}{g_{z}\mp z_{avg}}\right),$$

where $C_{\pm_{parallel}}$ is the capacitance for a parallel plate capacitor for for each of the rising and falling plates and $z_{avg}$ is the average displacement of both mass ends. Sensitivity calculations from the FEM simulation inner mass displacement results using both the tilted plate capacitor and the parallel plate capacitor formulas are shown in Table 3, and compared to the sensitivity value predicted by (26). The sensitivity reported is differential:

(29) $$S=\frac{\Delta C}{\Omega }=\frac{C_{+}-C_{-}}{\Omega }.$$

As can be seen, both the tilted capacitor and parallel plate capacitors yield the same result, and they go in close agreement with the sensitivity value predicted by the analytical model (26).

### 2.4. Device Fabrication

The fabrication of an X/Y- Gyroscope requires the tuning of out-of-plane with in-plane mechanical modes. In the case of single mass for drive and sense modes, this is done by optimizing the springs dimensions responsible for the in-plane and out-of-plane mechanical modes. However, there will be a clear trade-off between setting the Eigen frequencies of the in-plane and out-of-plane mechanical modes. Having two different heights for the device layer allows a degree of freedom to ease such trade-off.

Moreover, to improve the sensitivity’s for out-of-plane motion, we will need to have an additional electrode above or below the moving structure to sense/actuate in the out-of-plane direction. This will impose small gap between the additional electrode and the moving structure associated with a stopper to overcome any possible stiction.

In addition, the fabrication process should offer a good vacuum encapsulation to ensure high quality factor for the drive and sense mechanical modes. The vacuum level will need also to be stabilized and last for long period without possible internal out-gassing leading to the increase of the pressure and thus lower the quality factor due to the fluid damping mechanisms. Introducing a getter layer ensures the stability of the vacuum level achieved during the encapsulation process.

**Figure 11:**
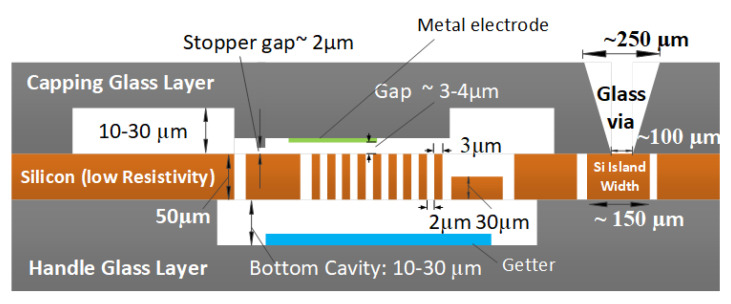
A cross-section for X/Y Gyroscope typical fabrication process.

Bulk micromachining processes on silicon using DRIE will be favorable to achieve good inertial mass and robust movable structures. Using Silicon on glass technologies allows good electrical insulation to overcomesubstrate capacitance [19]. Figure 11 illustrates a possible process implementation for an X/Y Gyroscope. One of the existing Silicon on glass fabrication processes that can be a starting point for our target process is the fabrication process offered by F-ENAS [20]. The original process was modified by F-ENAS to introduce the aforementioned features and fabricated the dedicated run in their facilities.

**Figure 12:**
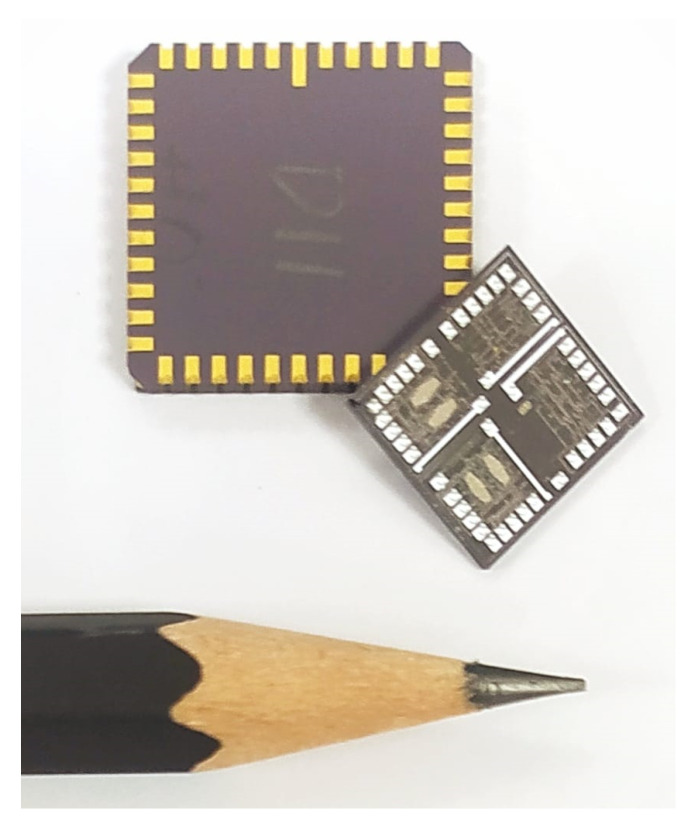
A picture of diced die and LCC packaged die.

The diced die contain four copies of the design and placed on orthogonal orientations to allow the testing of X and Y orientations. The diced dies from the fabrication runs were die-attached and wire-bonded in LCC package to streamline screening and testing. Figure 12 shows one of these dies before packaging. The die size is around 24 × 24 mm and contains four design variants per die.

## 3. Results

### 3.1. Interface Electronics

**Figure 13:**
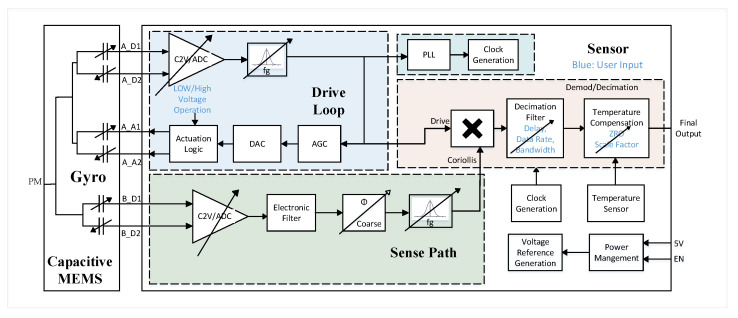
ASIC system consists of the sense path, the drive loop, and output decimation filter controlled by the user. In addition, a temperature sensor is included to compensate the interface through temperature compensation circuit.

The testing of gyroscopes requires a sophisticated interface electronics to capture the functionality and performance expected. An Application Specific ICs (ASIC) is used for this purpose. The ASIC uses a an analog-mixed signal ASIC with different blocks for readout, control, and compensation [21]. Figure 13 illustrates the block diagram of the ASIC. The ASIC considers the MEMS gyroscope as a capacitor with variable capacitance.

The analog circuits in the ASIC are the capacitance to voltage converter, Phased Locked Loop, power management, voltage reference, and clock generation. For low noise, and low power consumption most ASIC circuits were implemented on the digital domain when it is possible. Moreover, The digital implementation allows also generic operation with user programmable interface option to suit different gyroscope designs.

The ASIC consists of drive loop, sense loop, a clock generation block for the sense and drive loops, and decimation filter. The readout of the data is controlled through the decimation filter where data rate and bandwidth are programmable by the user. In addition, the electrodes of the MEMS gyroscope can be compensated by the user.

The drive loop is used to identify the MEMS gyroscope resonant frequency and drive it at this frequency with a certain voltage to translate any angular motion that may occur to the sense direction (Coriolis Force Concept). To identify the resonant frequency of the MEMS gyroscope, a feedback loop is established where the detected current on the drive detection electrode is converted to output analog voltage signal by a differential capacitance to voltage converter circuit (CVC) followed by ADC. The oscillation’s amplitude is controlled by the automatic amplitude control (AAC) circuit.

The capacitance to voltage converter circuit is implemented as switched capacitor circuit i.e., discrete time implementation to low noise frequency and offset suppression. The gain of the C2V could be set for drive loop configuration to minimize the measurement noise but it must be low for stability reasons.

The transfer function of the capacitive inertial MEMS exhibit a second order low-pass transfer response. That may suggest to use the MEMS as filtering elements in the drive loop since the MEMS serve as ΣΔ loop filter [22], resulting in a 2nd order electromechanical ΣΔ loop. However, due to increased electronic noise that results in reducing effective ΣΔ loop quantizer gain, an electronic filter is introduced on the loop to avoid resolution penalty [23]. An open-loop mode of operation is allowed in the sense loop, where the feedback signal is disconnected and the sense loop ADC provides digital readout for the sensor input.

The purpose of digital processing back-end is to filter the output of the ΣΔ modulator generated by sense and drive loop and demodulate the multiplied signal. The demodulation output is decimated using a programmable decimation filter. Table 4 summarizes the ASIC main features.

### 3.2. Test Setups and Results

#### 3.2.1. Gyroscope Characterization and Trimming

The main target of the gyroscope characterization and trimming phase is to fine-tune ASIC configurable parameters to get the best performance of the MEMS Gyroscope. Gyroscope scale factor is calculated by applying a calibrated angular velocity and reading the gyroscope output. After calculating the gyroscope scale factor, noise and sensitivity are calculated.

**Figure 14:**
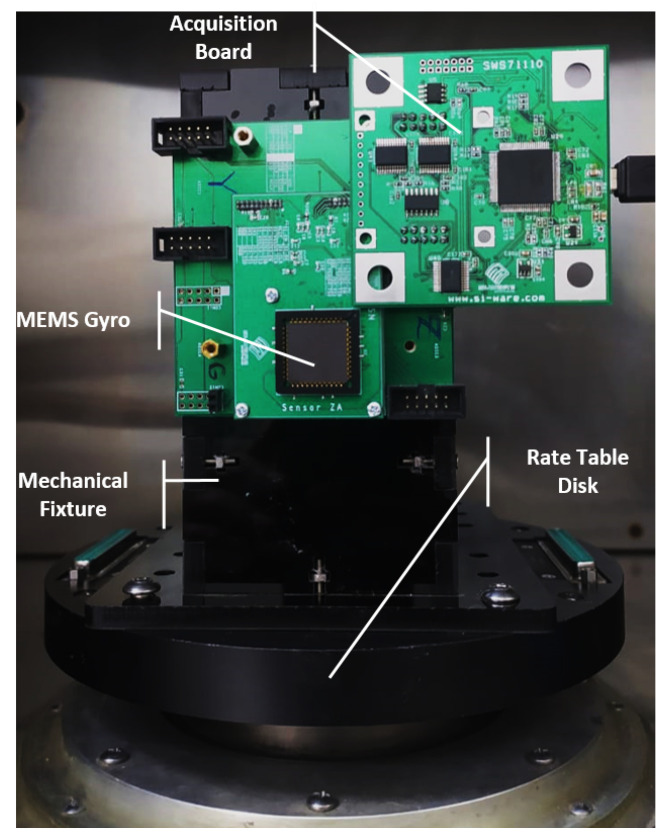
Experimental test setup. The fixture is used to transmit the angular velocity from the rate table disk to the attached ASIC/gyroscope interfacing Board. Acquisition broad is used to interface with ASIC.

The test setup, shown in Figure 14, which consists of a rate-table (Ideal-Aerosmith 1291BL Series) and SPI box (8451 NI), was built to measure Gyroscope performance. Gyro/ASIC interfacing board is used to provide electrical connectivity between Gyro MEMS and ASIC. ASIC uses two different communication protocols, SPI Protocol is used to get the gyroscope output reading, and the 2nd protocol is used to send the reading of gyroscope oscillation in drive and sense directions which are very useful during ASIC parameter tuning phase. A special data acquisition board is used to interface with ASIC through both SPI and the special communication protocol.

**Figure 15:**
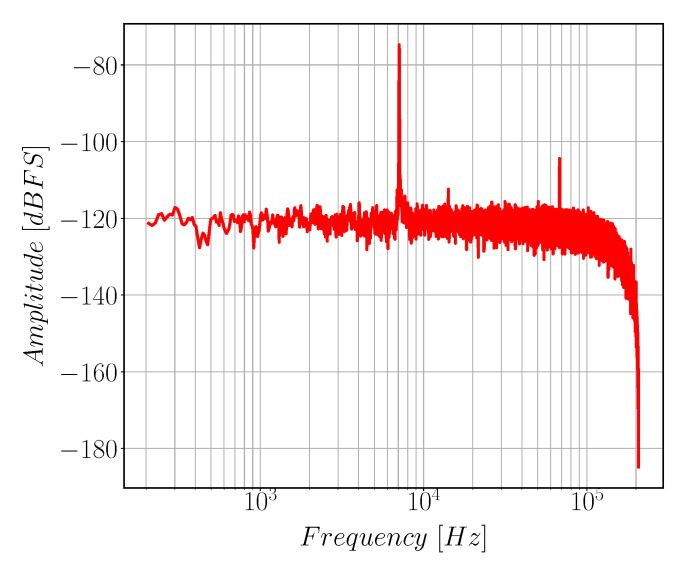
Frequency response of the drive mode of the gyroscope.

Gyroscope resonance frequency is measured by applying p-n sequence through drive actuation electrodes and the Gyro response is captured by the ASIC from drive detection electrodes. Then, the drive natural frequency is calculated using fast Fourier transform. Figure 15 shows the resonance characteristics of the gyroscope drive mode. The measured drive resonance frequency is equal to 7110 Hz, which is about 444 Hz less than the design value due to fabrication tolerances. The sense resonance characteristics can not be measured using the ASIC as we are using open loop sense configuration.

**Figure 16:**
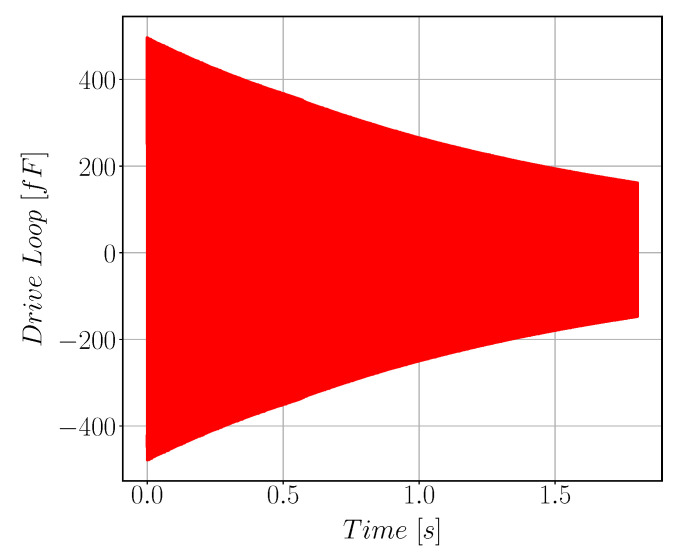
Exponentially decaying sinusoidal signal of the drive mode used for Quality Factor calculation. The measured QF is 33 k.

After we get a working and stable drive loop, drive mode quality factor is calculated using the logarithmic decay method which is done by switching off the drive actuation force and monitoring the rate of decay of the sinusoidal motion Figure 16 shows the decaying signal rate. Our gyroscope shows high drive-mode quality factor about 33k due to vacuum sealing.

**Figure 17:**
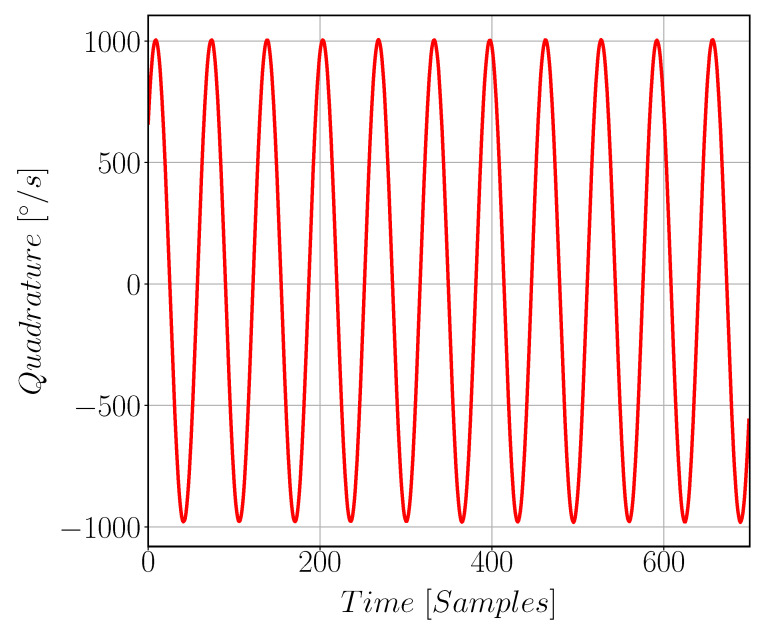
Quadrature signal in [°/s] which arises in the sense path of the gyroscope due to mechanical cross coupling between the drive and sense mode resulting from fabrication issues and the proof mass imbalance.

**Figure 18:**
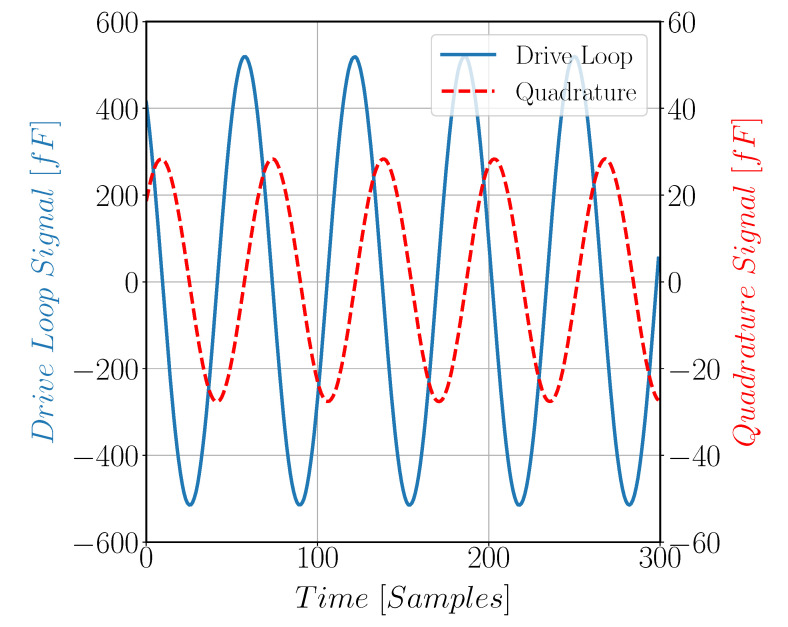
The proof mass motion in both the drive and sense directions at zero rate input velocity. The two signals were taken at different occasions.

**Figure 19:**
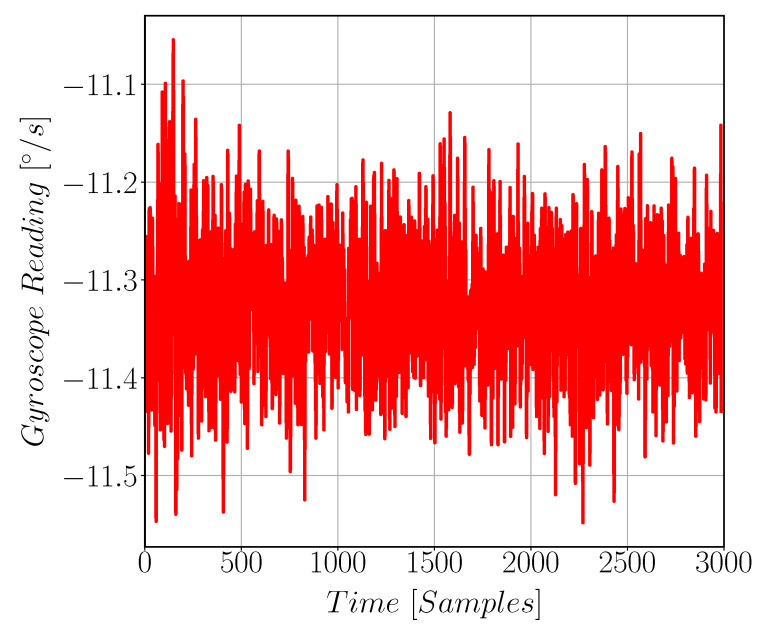
Raw Gyroscope reading while kept stationary. This represents the DC offset in the gyroscope reading. These data are processed to get the noise value for the gyroscope. R is Gyroscope reading in [°/s].

Figure 17 shows quadrature signal arises in Gyroscope Sense direction due to fabrication issues. Figure 18 compares the quadrature and the drive signals at zero input angular rate; note that the two signals have different scale. Gyroscope noise is found to be 4.5 m°/s/$\sqrt{\mathrm{Hz}}$ as shown in Figure 19. The measured parameters of the gyroscope, including sensitivity and quadrature, are reported in Table 5.

To compare the theoretical values obtained from the design equations and the measurement, we substituted with measured drive and sense frequencies, our theoretical model in (26) predicts the measured sensitivity with 98% accuracy.

#### 3.2.2. Allan Variance

**Figure 20:**
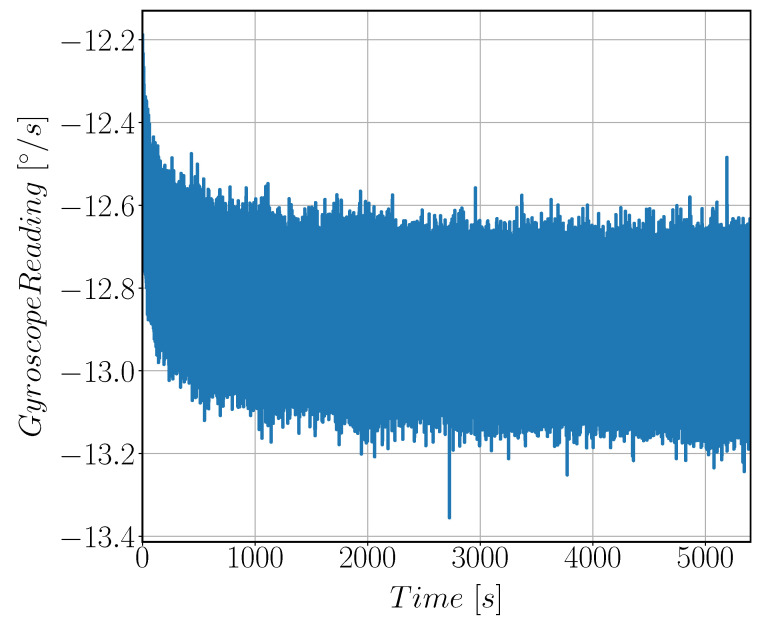
Raw sensor output signal in [°/s] at zero rate input velocity. The signal is acquired for 1.5 h and was used for Allan Variance calculations.

**Figure 21:**
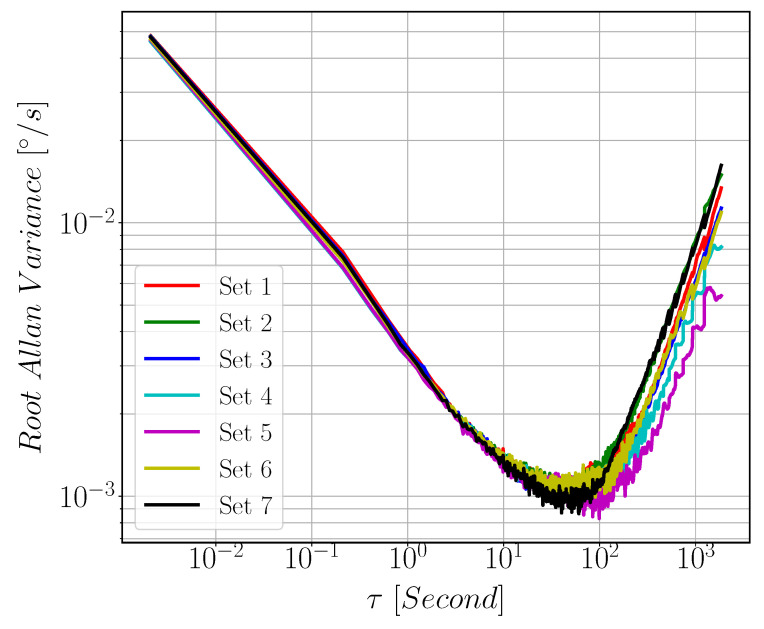
Allan Variance plot of the gyroscope obtained from 1 h drift data. τ is the averaging time in seconds. The minimum point of the curve occurs at τ = 100 s.

The target of this test is to measure bias-instability (BI) and angular random walk (ARW) of MEMS Gyroscope. This is done by acquiring zero rate sensor output signal for 1.5 h duration. Figure 20 shows the bias drift data acquired for 1.5 h at a sample rate of 500 Hz. We repeat this acquisition for several times with 0.5 h break between each iteration. The results are calculated for only 1 h data by neglecting the first 30 min data samples to avoid warm-up phase data. Measurements were done in temperature isolated environment to eliminate the effect of sensor output variations across temperature. 8451 NI-SPI Box was used to acquire Gyroscope reading sent by the ASIC through SPI Protocol. The Allan variance plots are illustrated in Figure 21. As we can see form the figure that the minimum point of the curve occurs at τ = 100 s. The Bias Instability (BI) and Angular Random Walk (ARW) are estimated from the figure to be 5.5 °/h and 0.2 °/$\sqrt{h}$, respectively.

#### 3.2.3. Scale Factor Non-Linearity (SFNL)

**Figure 22:**
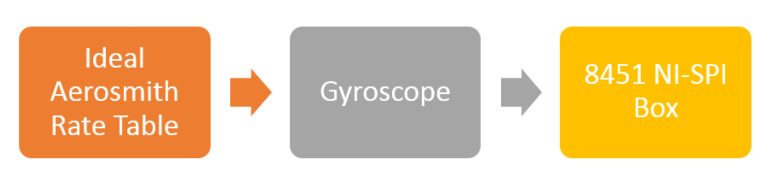
SFNL Test Block Diagram. SPI Box is used to acquire the Gyroscope reading sent from the ASIC through SPI protocol. Rate Table is used to apply angular velocity on the gyroscope.

**Figure 23:**
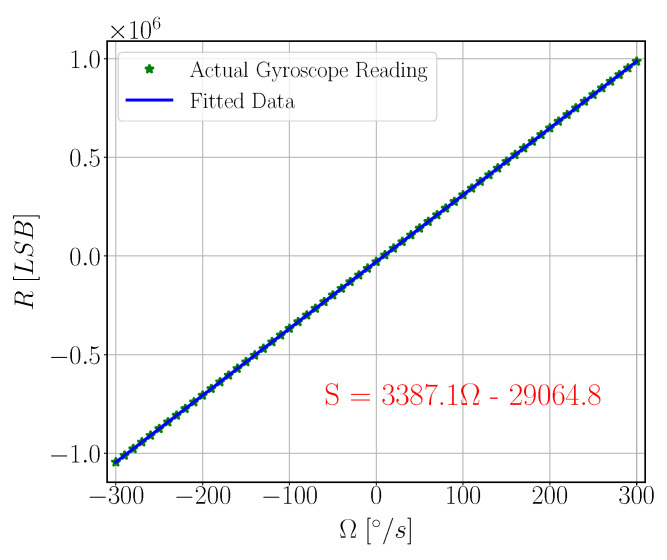
Scale Factor Non-Linearity. Ω is Angular Velocity input from the rate table in °/s, R is the Gyroscope reading in LSB.

In order to measure SFNL, the rate table was used to apply angular velocity input from −300 °/s to 300 °/s with step of 10 °/s. Gyroscope reading was acquired using 8451 NI-SPI Box. Figure 22 shows SFNL testing setup components. At each velocity step, 1200 samples of sensor output data and simultaneous rate table velocity are acquired then averaged. Using least square method, linear relation between rate table velocity and the gyroscope reading is calculated Figure 23.

**Figure 24:**
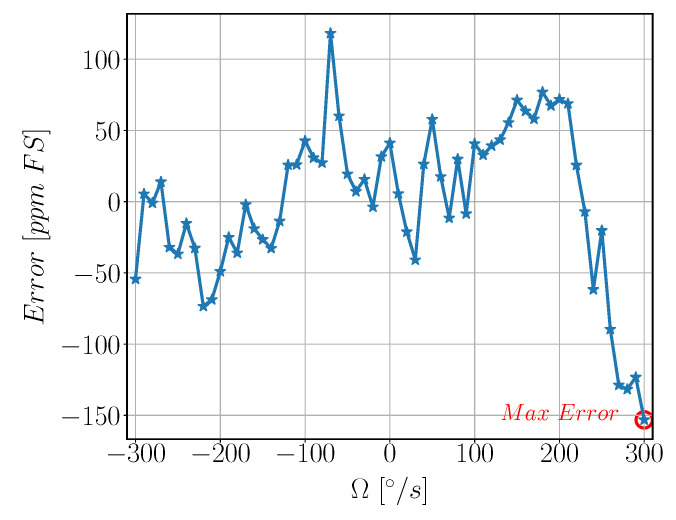
Measured velocity Error vs. Input Velocity. Ω is Angular Velocity input from the rate table in °/s, Error is error between the actual gyroscope reading and the corresponding theoretical output from linear relation calculated in part per million linearized with respect to full scale range [ppm FS].

The SFNL value for the gyroscope is the maximum error between the actual gyroscope reading and the fitted value calculated using least square method Figure 24. The SFNL is found to be 156 ppm FS.

## 4. Conclusions

In this paper, we present the design modeling, fabrication and testing of an electrostatic MEMS roll/pitch tuning fork gyroscope with in-plane drive and out-of-plane sense. We used a multi height DRIE process to enhance the decoupling between the drive and sense modes and hence limit the quadrature error. The gyroscope characterization was preformed using a dedicated ASIC for electronic interface of the gyrosocpe module. The experimental characterization of the gyroscope shows a good agreement between the analytical and FEM models with the measured results in terms of the mode ordering, the frequency split between the drive and sense modes, and its sensitivity. The angle random walk (ARW), bias instability, and full-scale (SFNL) were found experimentally as 0.2 °/$\sqrt{h}$, 5.5 °/h, and within 156 ppm FS, respectively.

## Figures and Tables

**Table 1:** Mode frequencies.

Mode	Frequency (Hz)
In-phase drive mode	7041
out-of-phase drive mode	7554
out-of-phase sense mode	8166
In-phase sense mode	9029

**Table 2:** Tolerance analysis of fabrication imperfection on the out-of-phase modes frequency separation.

Corner	Frequency Difference (Hz)
+100 nm	497
Normal design	612
−100 nm	737

**Table 3:** Inner mass displacement and corresponding sensitivity.

Parameter	Value
FEM inner mass ends displacements	52.5 and 32.5 pm
FEM inner mass ends displacement average	42.5 pm
FEM sensitivity by tilted plate capacitor formula	41.43 aF/dps
FEM sensitivity by parallel plate capacitor formula	41.43 aF/dps
Sensitivity predicted by (26)	43.45 aF/dps

**Table 4:** ASIC Features.

Parameter	Value
Drive Frequency [ kHz ]	1.9 to 50
Supply Voltage [V]	5
Input Noise [zF/$\sqrt{\mathrm{Hz}}$]	50
Temperature Range [°C]	−40 ot 85
ASIC Output Resolution [bit]	24
ADC Dynamic Range (in 100 Hz BW) [dB]	100
Output Filter BW (@400 kHz Sampling Freq.) [Hz]	500

**Table 5:** Main characterization results.

Parameter	Value
Measured comb travel distance	9.35 μm
Measured drive frequency	7110 Hz
Measured sense frequency	7942 Hz
Measured sensitivity	28.2 aF/°/s
Theoretical sensitivity by our model (26)	28.8 aF/°/s
Measured quadrature	1000 °/s
Noise	4.8 m°/s/$\sqrt{\mathrm{Hz}}$
Bias Instability	5.5 °/h
Angular Random Walk	0.2 °/$\sqrt{h}$

## Data Availability

Data in this paper is available from the corresponding authors upon request.
